# Adhering to our original aspiration, forging ahead with determination—Editor in Chief's new year address

**DOI:** 10.1002/ame2.70163

**Published:** 2026-02-16

**Authors:** Chuan Qin

**Affiliations:** ^1^ Chinese Association for Laboratory Animal Sciences Beijing China; ^2^ Institute of Laboratory Animal Sciences, Chinese Academy of Medical Sciences & Peking Union Medical College Beijing China

On the occasion of the New Year, I would like to extend my sincere gratitude and New Year greetings to the experts, scholars, author teams, and readers who have long supported the development of *Animal Models and Experimental Medicine* (*AMEM*). Over the past year, we have faced challenges together and achieved breakthroughs in academic influence, internationalization, and fulfilling social responsibilities. Looking ahead, we are filled with confidence as we strive to build an important bridge connecting laboratory animal science and technology with academic research.

## 1 CONTINUED STRENGTHENING OF DEVELOPMENT FOUNDATIONS

Over the past year, *AMEM* received over 550 submissions, marking a significant 40% increase from the previous year. The number of published articles also surged by 90%, reaching over 190. With these high‐quality academic outputs, *AMEM* successfully secured a place in **the Chinese Academy of Sciences (CAS) Journal Partition Q2 and maintained its position in JCR Q2**. The editorial board has now gathered 182 scholars from 20 countries, **with international members accounting for 47%**, forming a diverse team that combines disciplinary depth with geographical breadth. This team brings a global academic perspective and cutting‐edge research concepts to the journal. Meanwhile, the international reach of our authors and readers has also expanded, **with submitting authors from 53 countries this year**, an increase of 12 countries from the previous year, and reader full‐text views surpassing 300 000, further enhancing the journal's international academic influence.

## 2 DEEPENING THEMED SECTION PLANNING AND ACTIVELY PROMOTING IN‐DEPTH DIALOGUE IN CROSS‐DISCIPLINARY RESEARCH

This year, we have planned and launched several themed sections focusing on core issues in laboratory animal science and experimental medicine, such as “Frontier Exploration of Immunology and Cell Therapy,” “New Drugs for the Treatment of Epilepsy and Neurobehavioral Improvement,” “Ischemia–Reperfusion Injury,” “Frontiers in Neural Regeneration and Repair,” “Neurodegenerative Disease,” “The Role of Microbiota in Host Health,” and “Virus–Host Interactions.” These sections have sparked widespread and beneficial discussions. Additionally, we have provided **systematic coverage of the laboratory animal field in Asia**, offering an important window for the international community to understand the dynamics in this region.

**Laboratory animal replacement**, which concerns laboratory animal welfare and disciplinary development, **is a topic that the journal has organized special discussions on**, soliciting opinions from multiple stakeholders and actively facilitating dialogue among different viewpoints. Replacement is not only an inevitable requirement for adhering to animal welfare principles but also a crucial path for promoting sustainable development in the discipline. Although replacement is an eternal topic, it cannot be achieved overnight. It must be built on solid foundational research and gradually realized through technological innovation, which will be a direction that *AMEM* will continue to delve into in the future.

## 3 STAYING COMMITTED TO THE ACADEMIC MISSION AT HEART AND EXPANDING DIMENSIONS OF ACADEMIC EXCHANGE

While achieving success and fulfilling our academic mission, we recognize that hot topics serve as “wind vanes” for disciplinary development and deserve attention and timely focus. However, our journal firmly believes that **originality is the soul of academic research** and the lifeblood of the journal. We sincerely hope that more high‐quality research outcomes with originality, foresight, and scientific rigor will be submitted to our journal, filling academic gaps.

To better meet the publishing needs of the academic community and expand the space for academic exchange, *AMEM* will introduce several significant upgrades in 2026. In terms of publication scale, **the page count per issue will be optimized to 210 pages (up from 190 pages)** based on the journal's development characteristics and manuscript quality. The increased pages will solely be used to carry high‐quality original outcomes, providing a broader platform for academic display for our colleagues in the field. Regarding academic exchange and content construction, we plan to **organize international online and offline academic conferences**, breaking geographical barriers to gather global top experts for real‐time discussions. Building on our systematic coverage of the laboratory animal field in Asia this year, we will also **expand our regional research coverage to include countries and regions outside of Asia**, further promoting international academic exchange and mutual learning of experiences. Simultaneously, we **will launch the “Laboratory Animal Welfare: Theoretical & Technical Frontiers” themed section**, publishing multiperspective research outcomes and viewpoint commentaries to drive in‐depth development in related fields.

We look forward to continuing our collaboration with all colleagues, pooling our efforts, and jointly fostering academic prosperity in the fields of laboratory animal science and technology and experimental medicine!

